# Wound closure after endoscopic submucosal dissection of the colon using a novel detachable traction device: a rubber ring with a figure-of-eight sliding knot

**DOI:** 10.1055/a-2724-7905

**Published:** 2025-11-06

**Authors:** Jinpo Wang, Yunxin Chen, Miao Liu, Xiaoxiong Guo, Mingkai Zhuang, Zihan Chen, Fenglin Chen

**Affiliations:** 1 117890 Department of Gastroenterology, Fujian Medical University Union Hospital, Fuzhou, China; 2 117890 Fujian Clinical Research Center for Digestive System Tumors and Upper Gastrointestinal Diseases, Fujian Medical University Union Hospital, Fuzhou, China; 3 117890 Department of Gastrointestinal Endoscopy Nursing, Fujian Medical University Union Hospital, Fuzhou, China

Closure of post-endoscopic submucosal dissection (ESD) defects in the intestine remains challenging, often being time-consuming and incomplete. Previous studies reported a complete closure rate of only 76% following colorectal ESD 1 , with a pooled mean suturing time of 31.11 minutes 2 . Therefore, improved techniques for intestinal ESD wound closure are still being actively explored. Our team previously introduced the pre-placement sharp angle traction (PPSAT) method to assist ESD for colorectal laterally spreading tumors (LSTs) 3 . Building on this technique, we developed a novel device named SureLoop ( Fig. 1 ), which combines a rubber ring and a figure-of-eight sliding knot to facilitate both resection and wound closure ( Video 1 ).

**Fig. 1:**
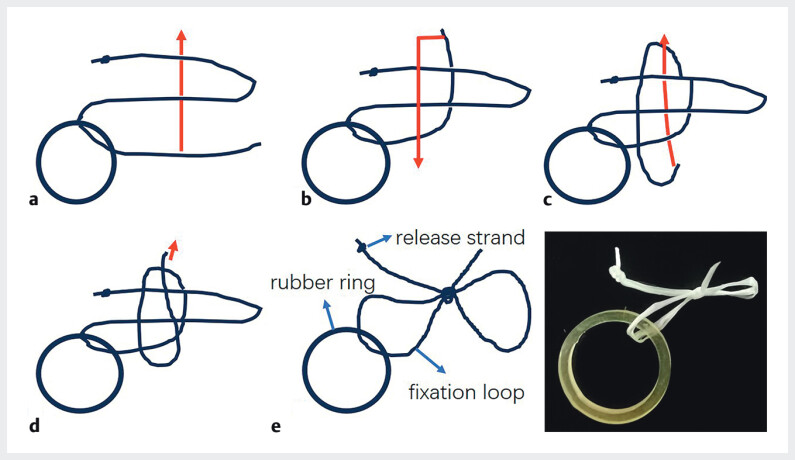
How to tie a “SureLoop” with floss and a rubber ring. **a** Create a loop by passing the end of the thread through from the back down. **b** Move it from the front to the lower left. **c** Pass it through the top left loop from the back. **d** Finally, tighten the thread at the end and the top of the lower left loop. **e** . Completed.

The novel SureLoop traction device was utilized to facilitate traction-assisted dissection and subsequent wound closure.Video 1

An LST measuring approximately 20 mm × 25 mm was identified in the rectum. After submucosal injection and circumferential incision, the SureLoop was anchored at an optimal position opposite to the lesion. The rubber ring was fixed on the anal side of the lesion using a clip, enabling effective traction via PPSAT technique to facilitate en bloc resection ( Fig. 2 **a** ). The fixation loop of the figure-of-eight knot was then clipped to one edge of the wound and pulled diagonally toward the opposite side, achieving sequential button-type closure ( Fig. 2 **b** ). After successful closure, the release strand of the knot was pulled with a clip or forceps to loosen and remove the knot ( Fig. 2 **c, d** ). Notably, a single rubber ring can accommodate two or more figure-of-eight knots if needed.

**Fig. 2:**
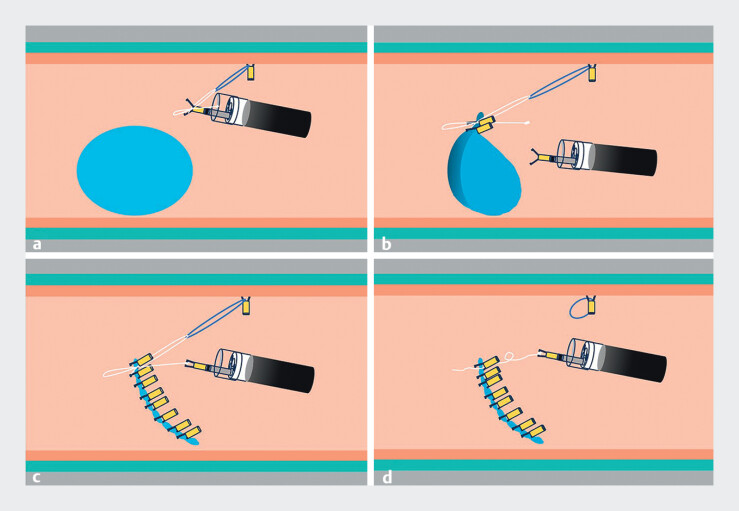
Schematic description of the strategy. **a** The SureLoop's rubber band is anchored at an appropriate distance and direction diagonally opposite to the lesion. An additional clip is used to secure the fixation knot of the figure-of-eight ligature on one side of the wound. **b** The wound edge is grasped and pulled toward the diagonally opposite side, facilitating easier, more precise, and rapid wound closure. **c** After complete wound closure, the release strand of the figure-of-eight ligature is engaged and gently pulled using a clip or forceps to undo the knot. **d** Successful wound closure is achieved. And the clip that loosens the knot can be repurposed to strengthen the wound.

During colorectal ESD, parallel alignment between the endoscope and the colon wall often complicates clip placement. The SureLoop device facilitates effective traction toward the anal side, reorienting the wound to face the endoscope directly, thereby simplifying the closure process, improving accuracy, and reducing both procedure time and number of clips required. We believe this device holds significant clinical promise, though prospective studies are warranted to validate its efficacy.

Endoscopy_UCTN_Code_TTT_1AQ_2AB

## Notes

Endoscopy E-Videos https://eref.thieme.de/e-videos
*E-Videos* is an open access online section of the journal *Endoscopy* , reporting on interesting cases and new techniques in gastroenterological endoscopy. All papers include a high-quality video and are published with a Creative Commons CC-BY license. Endoscopy E-Videos qualify for HINARI discounts and waivers and eligibility is automatically checked during the submission process. We grant 100% waivers to articles whose corresponding authors are based in Group A countries and 50% waivers to those who are based in Group B countries as classified by Research4Life (see: https://www.research4life.org/access/eligibility/ ). This section has its own submission website at https://mc.manuscriptcentral.com/e-videos .
